# Modelling the distribution and transmission intensity of lymphatic filariasis in sub-Saharan Africa prior to scaling up interventions: integrated use of geostatistical and mathematical modelling

**DOI:** 10.1186/s13071-015-1166-x

**Published:** 2015-10-24

**Authors:** Paula Moraga, Jorge Cano, Rebecca F. Baggaley, John O. Gyapong, Sammy M. Njenga, Birgit Nikolay, Emmanuel Davies, Maria P. Rebollo, Rachel L. Pullan, Moses J. Bockarie, T. Déirdre Hollingsworth, Manoj Gambhir, Simon J. Brooker

**Affiliations:** Faculty of Infectious and Tropical Diseases, London School of Hygiene & Tropical Medicine, London, UK; Faculty of Epidemiology and Population Health, London School of Hygiene & Tropical Medicine, London, UK; School of Public Health, College of Health Sciences, University of Ghana, Legon, Accra Ghana; Eastern and Southern Africa Centre of International Parasite Control, Kenya Medical Research Institute, Nairobi, Kenya; Ministry of Health, Abuja, Nigeria; NTD Support Center, Task Force for Global Health, Emory University, Atlanta, USA; Department of Vector Biology, Liverpool School for Tropical Medicine, Liverpool, UK; Warwick Infectious Disease Epidemiology Research, Warwick Mathematics Institute, University of Warwick, Coventry, UK; School of Life Sciences, University of Warwick, Coventry, UK; Department of Epidemiology and Preventive Medicine, Monash University, Melbourne, Australia

**Keywords:** Lymphatic filariasis, *Wuchereria bancrofti*, Bayesian geostatistical modelling, Mathematical modelling, Basic reproductive number, Sub-Saharan Africa

## Abstract

**Background:**

Lymphatic filariasis (LF) is one of the neglected tropical diseases targeted for global elimination. The ability to interrupt transmission is, partly, influenced by the underlying intensity of transmission and its geographical variation. This information can also help guide the design of targeted surveillance activities. The present study uses a combination of geostatistical and mathematical modelling to predict the prevalence and transmission intensity of LF prior to the implementation of large-scale control in sub-Saharan Africa.

**Methods:**

A systematic search of the literature was undertaken to identify surveys on the prevalence of *Wuchereria bancrofti* microfilaraemia (mf), based on blood smears, and on the prevalence of antigenaemia, based on the use of an immuno-chromatographic card test (ICT). Using a suite of environmental and demographic data, spatiotemporal multivariate models were fitted separately for mf prevalence and ICT-based prevalence within a Bayesian framework and used to make predictions for non-sampled areas. Maps of the dominant vector species of LF were also developed. The maps of predicted prevalence and vector distribution were linked to mathematical models of the transmission dynamics of LF to infer the intensity of transmission, quantified by the basic reproductive number (R_0_).

**Results:**

The literature search identified 1267 surveys that provide suitable data on the prevalence of mf and 2817 surveys that report the prevalence of antigenaemia. Distinct spatial predictions arose from the models for mf prevalence and ICT-based prevalence, with a wider geographical distribution when using ICT-based data. The vector distribution maps demonstrated the spatial variation of LF vector species. Mathematical modelling showed that the reproduction number (R_0_) estimates vary from 2.7 to 30, with large variations between and within regions.

**Conclusions:**

LF transmission is highly heterogeneous, and the developed maps can help guide intervention, monitoring and surveillance strategies as countries progress towards LF elimination.

**Electronic supplementary material:**

The online version of this article (doi:10.1186/s13071-015-1166-x) contains supplementary material, which is available to authorized users.

## Background

Lymphatic filariasis (LF) is a mosquito-borne disease caused by the filarial worms, *Wuchereria bancrofti*, *Brugia malayi* and *B. timori*. Since the launch of the Global Programme to Eliminate Lymphatic Filariasis in 2000 an estimated total of 975 million people, including 198 million in sub-Saharan Africa, have benefitted from mass drug administration (MDA) programmes that deliver antifilarial drugs [1]. As a result of these efforts, it is estimated that the global prevalence of infection has decreased by 30 % and 18.2 million cases of LF morbidity have been averted [1]. At a country level, an increasing number of national LF control programmes have completed five or more rounds of MDA and, as a consequence, are conducting transmission assessment surveys (TAS) to determine whether prevalence of LF is <1 % and MDA can stop [2]. After stopping MDA, programmes will still need to conduct surveillance to ensure transmission has not re-emerged (i.e. there has been no recrudescence). This can either be achieved by periodic surveys, for example, by repeating a TAS 2–3 years after stopping MDA, or through screening of routine blood samples [3]. For the process of verification, countries will also need to conduct surveillance in areas judged to be non-endemic at the start of the programme. To help reduce costs, surveillance can be stratified according to the risk of recrudescence. This risk may be predicted from analysis of the historical, pre-intervention transmission levels, vector type and capacity and environmental and demographic factors known to influence the intrinsic sensitivity (receptivity) of transmission [4–6]. Recent work at the country level highlights the environmental, socio-demographic, and intervention drivers of LF and how this information can be used to stratify areas according to likelihood of transmission being interrupted or persisting [7–9]. Other work has used Bayesian geostatistical modelling to predict the distribution of LF at country [10] and continental [11] scales.

In this paper, we use a combination of Bayesian geostatistical and mathematical modelling to develop maps of the prevalence and transmission intensity of bancroftian filariasis in sub-Saharan Africa (SSA) prior to large-scale control. We use these maps to inform a stratified approach to LF surveillance. The work builds on recent work to develop a global atlas of LF infection [12], conducted as part of the *Global Atlas of Helminth Infections* project (www.thiswormyworld.org) [13].

## Methods

### Data sources and data selection

Data on the prevalence of LF infection were identified from searches of the formal and informal literature and direct communication with LF control programmes. Details of the search strategies, inclusion criteria, data abstraction and geolocation procedures are provided by Cano et al. [12]. In brief, only population-based survey data based on random sampling were included, whereas data from non-random data, including surveys from hospitals, prisons, mental institutions or military facilities, were excluded. In the current analysis, only surveys conducted prior to the implementation of countrywide, population-based MDA were included. Infection prevalence was defined as either (i) the proportion of surveyed individuals with detectable microfilaraemia or (ii) the proportion of surveyed individuals with detectable antigenaemia. A table with a brief description of the surveys compiled and used in this work is provided as supplemental information (see Additional file 1).

Microfilaraemia (mf) was estimated from examination of thick blood films collected during night blood surveys to detect the presence of microfilariae using microscopy [14, 15]. It is generally assumed that the sensitivity of methods for detecting microfilaraemia is influenced by the volume of blood sampled and previous authors have adjusted prevalence estimates according to blood volume [16]. We therefore investigated this issue further but found that although estimates of infection prevalence varied according to blood volume, we were unable to derive consistent adjustment factors and therefore did not adjust prevalence estimates according to blood volume (see Additional file 1). It is also known that concentration and counting chamber methods have greater sensitivity [17] and therefore where estimates were derived using both concentration methods and thick blood smears (*n* = 10 surveys), we used only the thick smear data.

*Wuchereria bancrofti* antigenaemia was typically estimated using an immuno-chromatographic card test (ICT) [14, 18]. These tests are more sensitive than mf detection and can be conducted on blood collected at any time of the day and therefore since 2000 have been the diagnostic method of choice for mapping the distribution of LF caused by *W. bancrofti*. Recent work has highlighted potential cross-reactivity of the ICT test with *Loa loa* [19], therefore we excluded ICT-based surveys conducted in areas of *L. loa* transmission (*n* = 314), as defined by Zouré et al. [20].

**Fig. 1:**
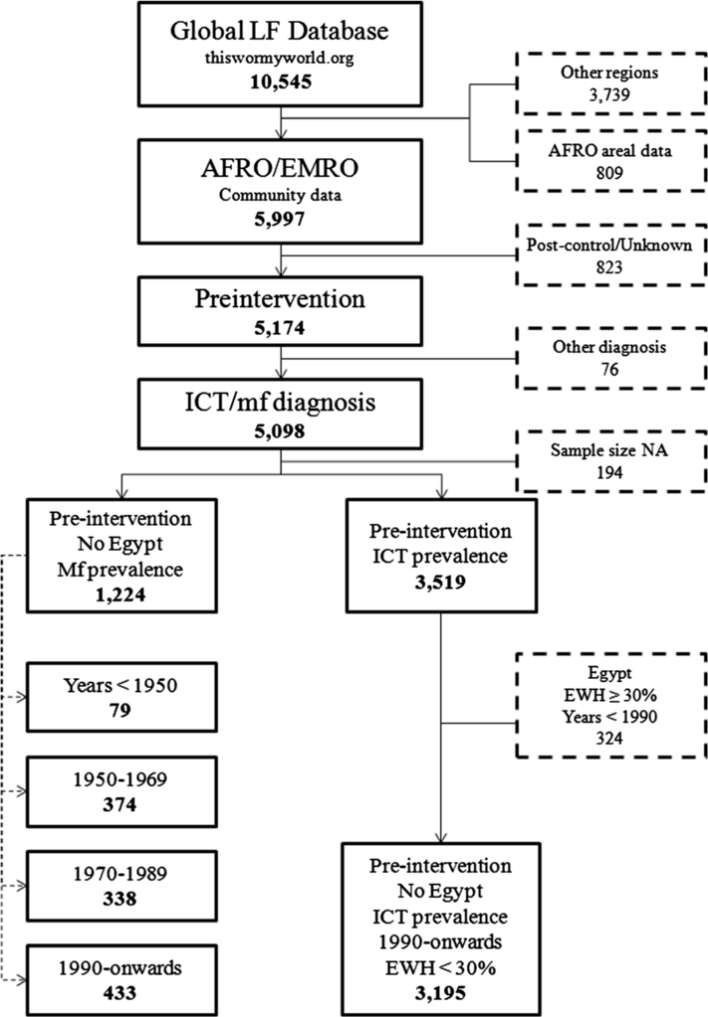
Selection of data for inclusion into the modelling, based on sample size, diagnostic method. NA: not available; EWH: eye worm history (presumptive of loiasis)

**Fig. 2:**
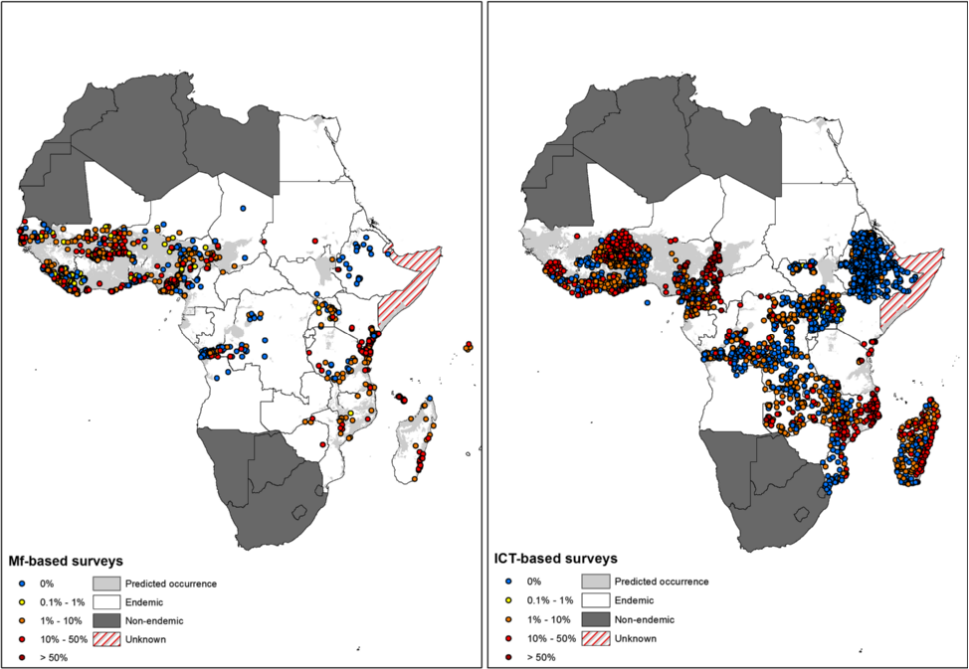
The spatial distribution of data on the prevalence of microfilaraemia (mf) (*n* = 1217 surveys) and antigenaemia, based on immuno-chromatographic card test (ICT) data (*n* = 3197 surveys). Countries defined as non-endemic by the World Organization are shown. Also shown are the spatial limits of transmission as previously defined by Cano et al*.* [12]

Data derived from parasitological blood examinations during day time (*n* = 71), ELISA (*n* = 70), clinical examinations (*n* = 24) and other molecular or antibody-based tests (i.e. PCR, IFI, new rapid test) (*n* = 17) were excluded because of the lack of comparable data. Finally, we excluded data from Egypt since this country is no longer considered endemic for LF and has a different transmission ecology from SSA [21, 22]. Figure 1 summarises the survey data selection and Fig. 2 shows the geographical distribution of survey data by diagnostic method.

### Covariate variables

There are a number of climatic, environmental and demographic factors that influence the transmission and epidemiology of LF, including temperature, humidity, elevation and population density [12, 23–25]. We generated a suite of climatic and environmental covariates from surfaces interpolated from meteorological stations or remote sensing imagery, including estimates of mean, minimum and maximum temperature and precipitation at 1 km^2^ resolution [26], averaged long-term estimates of land surface temperature [27], elevation at 1 km^2^ resolution [28], and aridity index [28], averaged enhanced vegetation index for the period 2000 to 2012 [27], and land cover data [29]. Estimates of population density were obtained from the Gridded Population of the World [30] and the United Nations Environment Programme [31], which we used to classify areas as urban, peri-urban or rural areas, based on the assumption that urban extents (UE) have a population densities ≥1000 persons/km^2^, peri-urban >250 persons/km^2^ within a 15 km distance from the UE edge, and rural <250 persons/km^2^ and/or >15 km from the UE edge. From these datasets of population density, population growth rate for the period 1960 to 2010, as a measure of the change rate in population over this period, was also calculated. A detailed description of each covariate and source is provided in the supplemental file (Additional file 2: Table S3). Each covariate grid was resampled to a 10 km spatial resolution using bilinear interpolation for continuous surface and a majority approach for categorical data [32]. Covariate data were extracted to survey locations using ArcGIS Desktop v10.2 (Environmental Systems Research Institute Inc., Redlands CA, USA).

### Vector distribution maps

LF is transmitted mainly by mosquito species belonging to the *Anopheles, Culex,* and *Mansonia* genera and to lesser extent *Aedes*, *Coquillettidia* and *Ochlerotatus* genera [33, 34]. *Anopheles* mosquitoes are the main vectors of LF through much of west and central Africa and inland east Africa, whereas *Culex* species are the main vectors in coastal east and southern Africa [33]. Studies have shown that the survival, parasite uptake and development of infective L3 stages and overall transmission potential varies by vector species [35–37]. In order to capture these species differences, we developed maps of the distribution of each dominant LF vector species: *Anopheles, Culex* and *Mansonia*.

**Fig. 3:**
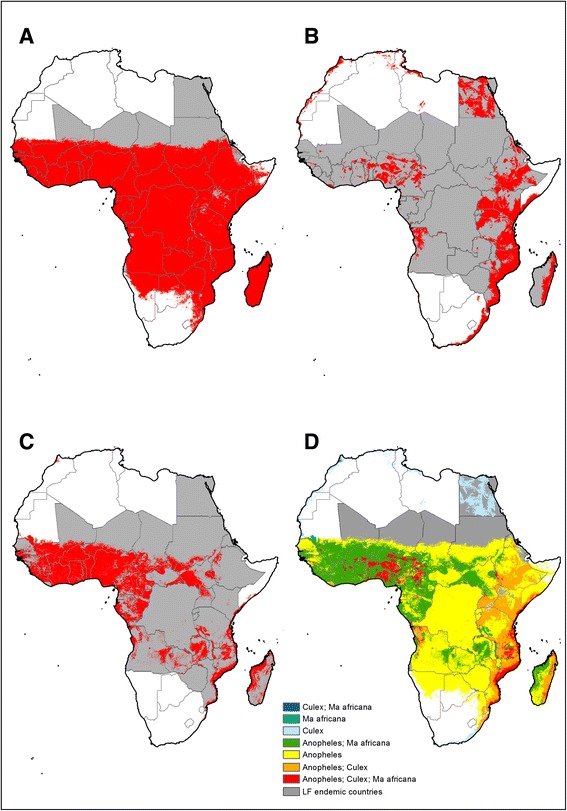
Predicted occurrence of the major potential vectors of lymphatic filariasis: **a** *Anopheles*, **b** *Culex*, **c** *Mansonia*, and **d** overlap of species

Maps of the distribution of mosquitoes belonging to the *An. gambiae* complex and *An. funestus* complex were obtained from the Malaria Atlas Project project (http://www.map.ox.ac.uk/) [38, 39] and a binary map displaying the distribution of each complex was created. Maps of *Culex* and *Mansonia* mosquitoes were obtained from the VectorMap project (http://www.vectormap.org/), which collates collection records of major vector insects and uses maximum entropy ecological niche modelling to develop occurrence maps [40, 41]. The maps present the probability of occurrence of *Cx. quinquefasciatus, Cx. pipiens, Cx. univittatus,* and *Mansonia Africana*, and we used a 90 % probability threshold to indicate dominance of a particular species according to a set of presence records obtained from the Global Biodiversity Information Facility database [42]. Each of the species-specific binary maps were combined to produce gridded maps of mosquito distribution by genus which, in turn, were combined into a single map of the different LF vector species at a 5 km spatial resolution (Fig. 3). The developed maps of *Anopheles, Culex* and *Mansonia* genera were subsequently incorporated as covariates in the goestatistical and mathematical modelling.

### Geostatistical modelling approach

The prevalence of mf and prevalence of antigenaemia were modelled separately due to a poor correlation between outcomes and the inability to predict one from another, as detailed elsewhere [43]. Bayesian geostatistical models, which included both fixed and random effects, were used to predict the spatial distribution of each outcome. Fixed effects quantify the effects of the covariates on LF infections, whereas random effects account for unexplained spatial variation whose structure can eventually help identify anomalous areas of high or low risk that can be further investigated.

The ICT-based model accounted for environmental and demographic covariates and spatial random effects as data spanned only 15 years and no temporal trend was observed in the data. From ICT-based data, we fitted a model in which conditional on the true prevalence *P*(*x*_*i*_) at location *x*_*i*_, *i* = 1, …, *n*, the number of positive results *Y*_*i*_ out of *N*_*i*_ individuals sampled at location *x*_*i*_, follows a binomial distribution *Y*_*i*_*|P* (*x*_*i*_) *~ Binomial(N*_*i*_*,P* (*x*_*i*_)). The log-odds of *P*(*x*_*i*_) is modelled as logit(*P*(*x*_*i*_)) = *z*_*i*_*β* + *S*(*x*_*i*_) + *u*_*i*_, where *z*_*i*_ = (1, *z*_*i*1_, …, *z*_*ip*_) denotes the vector of the intercept and the *p* covariates considered, and *β* = (*β*0, *β*1, . . ., *β*p)*’* is the coefficient vector. *S*(*x*_*i*_) is a zero-mean Gaussian process with Matérn covariance function that represents any residual spatial variation which is not explained by included covariates. The Mátern covariance function is expressed as

$$ \mathrm{C}\mathrm{o}\mathrm{v}\left(S\left({x}_i\right),S\left({x}_j\right)\right) = \frac{\upsigma^2}{2^{\upsilon -1}\Gamma \left(\upsilon \right)}{\left(k\parallel {x}_i-{\mathrm{x}}_{\mathrm{j}}\parallel \right)}^{\upsilon }{K}_{\upsilon}\left(k\parallel {x}_i-{\mathrm{x}}_{\mathrm{j}}\parallel \right), $$

where *Κ*_*υ*_ is the modified Bessel function of second kind and order *υ* > 0. The integer value of *υ* determines the mean square differentiability of the underlying process. *υ* is usually fixed since typically it is poorly identified in applications. *σ*^2^ is the marginal variance and *k* > 0 is a scaling parameter related to the range *ρ*, the distance at which the *S*(*x*_*i*_) and *S*(*x*_*j*_) are almost independent. In particular, we used the empirically derived definition $\rho = \sqrt{8\upupsilon}/k$, with *ρ* corresponding to the distance at which the spatial correlation is approximately 0.1. We also include in the model a spatially unstructured random effect, *u*_*i*_, that captures the effects of unmeasured characteristics that affectall members in the survey. *u*_*i*_ are assumed to be independent zero-mean Gaussian distributed with precision *τ*_*u*_.

The model for mf prevalence incorporated a temporal fixed effect to capture changes in mf prevalence over time and a spatio-temporal random effect under the assumption that fitted temporal correlations exist only with the preceding year [44]. Here, the number of positive results *Y*_*it*_ out of *N*_*it*_ people sampled at location *x*_*i*_, *i* = 1, …, *n* and time *t* = 1, 2, 3, follows a binomial distribution

$$ {Y}_{it}\Big|P\left({x}_i,t\right)\sim Binomial\left({N}_{it},P\left({x}_i,t\right)\right), $$

$$ logit\left(P\left({x}_i,t\right)\right)={z}_{it}\beta +\xi \left({x}_i,t\right)+{u}_{it}, $$

Here *ξ*(*x*_*i*_, *t*) denotes the between-location-time random effect at location *x*_*i*_ and time *t.*

$$ \xi \left({x}_i,t\right)=a\xi \left({x}_i,t-1\right)+w\left({x}_i,t\right), $$

where |*a*| <1, and *ξ*(*x*_*i*_, 1) follows the stationary distribution of a first-order autoregressive process, namely N(0, σ_w_^2^/(1 − *a*^2^)). *W*(*x*_*i*_, *t*) is a spatial correlation term. Each *w*(*x*_*i*_, *t*) follows a zero-mean Gaussian distribution. The *w*(*x*_*i*_, *t*) is temporally independent but spatially dependent at each time t, with Matérn covariance function. *u*_*it*_ denotes the unstructured random effect and are assumed to be independent zero-mean Gaussian distributed with precision *τ*_*u*_.

### Variable selection and model development

We followed a model selection procedure to identify an optimal suite of covariates to include in the fixed effects part of the geostatistical models. In order to reduce any potential collinearity and confounding effects, we first grouped the variables and use a formal model selection criterion to select one variable within each of the groups (Additional file 2: Table S4). Continuous variables with an absolute value of correlation coefficient higher than 0.8, were part of the same group. Land cover categories formed another group. Within each group, we investigated the relationship between infection prevalence and each potential explanatory variable by fitting univariate generalized linear models relating the logit of infection to each of the variables (Additional file 2: Table S5). We compared the univariate models in terms of the Akaike Information Criterion (AIC) and selected the variables which had the lowest AIC in the univariate analysis. AIC is defined as

$$ \mathrm{A}\mathrm{I}\mathrm{C} = -2l\left(\widehat{\theta}\right) + 2\mathrm{k}, $$

where $l\left(\widehat{\theta}\right)$ is the maximum log-likelihood function and *k* is the number of parameters. After that, we explored further simplification of the model by backward elimination of selected variables until it was no longer possible to reduce AIC by elimination of any of the remaining variables.

The age group (either below or above 15 years of age) of individuals studied on the prevalence surveys was considered at every step of the modelling process. In addition, population density was considered for the periods at which the surveys were undertaken. Since ICT-based data were available for surveys conducted from 1990 onwards, estimates of population density at 2000 were used. For mf data, in order to take into account temporal changes in population density, gridded maps of population density at 1960, 1980 and 2000 were used accordingly with the period of survey (1950–1969, 1970–1989, 1990–onwards).

The selection of covariates at this stage in the overall model-fitting process ignores spatial and temporal correlation, and as a result is likely to over-state the statistical significance of covariate effects. In the final stage of the model-fitting process we re-assess the covariate effects and their significance within a spatial mixed model for the ICT data and a spatiotemporal mixed model for the Mf data that take into account the spatial and spatiotemporal correlation, respectively.

### Model validation

We assessed the predictive ability of the model using a leave-one-out cross-validation procedure. In this approach, a single observation is retained as the validation data, and the spatial model is fitted to the remaining data. Then, the observation in the validation data is predicted using the fitted model. The validation data needs to spatially represent the whole region where the prevalence is predicted. Therefore, instead of repeating the cross-validation procedure using each observation once as the validation data, we used each of the locations of a spatially representative sample of the prediction surface. To obtain a valid data set, 20 % of the observations were sampled without replacement where each observation had a probability of selection proportional to the area of the Thiessen polygon surrounding its location, that is, the area closest to the location relative to the surrounding points.

The performance of the model was assessed by comparing the observed and the predicted prevalences at each location, and by calculating the coverage probabilities of 95 % confidence interval (CI), that is, the proportion of times that the observed prevalence rates are within 95 % CIs. Specifically, we computed the correlation between the predicted and the observed prevalences, the mean prediction error (ME) defined as

$$ \mathrm{ME}=\frac{1}{\mathrm{m}}{\displaystyle \sum_{\mathrm{i}=1}^{\mathrm{m}}}\left({\mathrm{p}}^{*}\left({\mathrm{x}}_{\mathrm{i}}\right)-\mathrm{p}\left({\mathrm{x}}_{\mathrm{i}}\right)\right), $$

and the mean absolute error (MAE) defined as

$$ \mathrm{M}\mathrm{A}\mathrm{E}=\frac{1}{\mathrm{m}}{\displaystyle \sum_{\mathrm{i}=1}^{\mathrm{m}}}\left|{\mathrm{p}}^{*}\left({\mathrm{x}}_{\mathrm{i}}\right)-\mathrm{p}\left({\mathrm{x}}_{\mathrm{i}}\right)\right|, $$

where m is the number of observations in the validation data, p*(x_i_) is the predicted prevalence, and p(x_i_) is the observed prevalence at location x_i_, i = 1, …, m.

### Implementation and spatial prediction

The models were fitted using the Integrated Nested Laplace Approximation (INLA) [45, 46] and the Stochastic Partial Differential Equation (SPDE) [47] approaches. INLA is a computationally less-intensive alternative to MCMC designed to perform fast Bayesian inference on a large class of latent Gaussian models. By using a combination of analytical approximation and numerical integration, INLA allows to fit models represented as follows,

$$ \left\langle {Y}_i|S,\theta \right\rangle \sim p\left\langle {Y}_i|{\eta}_i,\theta \right\rangle, $$

$$ {\eta}_i={\displaystyle \sum_j{c}_{ij}}{S}_j, $$

$$ \left\langle S|\theta \right\rangle \sim N\left(0,Q{\left(\theta \right)}^{-1}\right), $$

$$ \left(\theta \right)\sim p\left(\theta \right), $$

where *Y* denotes the observation variable that assume independence conditional on some underlying latent Gaussian field *S* and a vector of hyperparameters *θ*, and *η* is a linear predictor based on known covariate values *c*_*ij*_. The analysis of spatial point process data is possible by combining INLA and the SPDE approach. This consists in representing the continuously indexed Gaussian field *S*(*x*), as a discretely indexed Gaussian Markov random field (GMRF) by means of a basis function representation defined on a triangulation of the domain of interest. Thus,

$$ S(x)={\displaystyle \sum_{g=1}^G{\psi}_g}(x){S}_g, $$

where *G* is the number of vertices in the triangulation, *ψ*_*g*_(⋅) are piecewise polynomial basis functions on each triangle, and {*S*_*g*_} are zero-mean Gaussian distributed weights. The INLA and SPDE approaches are easily applied thanks to their implementation in the R-INLA software package [5].

We assigned a flat improper prior for the intercept, ${\beta}_0\sim N\left(0,{\tau}_{\beta_0}^{-1}\right)$ with ${\tau}_{\beta_0}=0$, and independent vague Gaussian priors with fixed precision for all other components of the fixed effects, *β*_*i*_ ∼ *N*(0, 1/0.001), *i* = 1, … *p*. The smoothness parameter *ν* was considered fixed to 1 implying a continuous domain Markov field. In the spatial model, the vector of weights *S* = (*S*_1,._.., *S*_*G*_)′ is assigned a Gaussian distribution, *S* ∼ *N*(0, *Q*^− 1^) where *Q* is a sparse precision matrix depending on the Matérn covariance function parameter *κ* and variance *σ*^2^. In the spatiotemporal model, for *ξ* = (*ξ*_1_, …, *ξ*_*n*_) ', we use a Gaussian prior with zero mean and precision matrix depending on the autocorrelation hyperparameter *a, k* and σ_w_^2^. To complete the models, we assign *τ*_*u*_ a vague Gamma prior.

The model output provided the posterior distribution for each of the model parameters. We summarized these distributions to obtain the posterior mean and 95 % CI of the fixed effects and hyperparameters. Predictions of infection prevalence were provided at a spatial resolution of 10 km. Maps showing the predicted LF prevalence as well as its uncertainty were generated by summarizing the posterior distributions of the prevalence obtained at each of the prediction locations. Specifically, the predicted prevalence was represented as the posterior mean, and its uncertainty was represented using the 95 % CI. Predictions were standardized to provide estimates for entire communities (children and adults combined) and for the period 2000-onwards.

### Mathematical modelling of the intensity of LF transmission

The prevalence of LF infection provides a useful metric to guide the planning of control, but provides limited insight to the dynamics of transmission. Instead the intensity of infection transmission is best quantified by the basic reproduction number, R_0_, which for macroparasites such as LF can be defined as the average number of female offspring per adult female worm surviving to reproduction (in the absence of density-dependence i.e. phenomena such as host immunity or worm mating probability, which accelerate or curtail the production of parasite life stages) [48]. Existing analytic expressions for R_0_ for LF, and helminths with similar natural history such as onchocerciasis, are defined in terms of average worm burden or microfilarial load in a community [49–51], yet the majority of LF studies only present data on the prevalence of infection. In the framework published by Gambhir et al. a simple, differential equation transmission model of LF is used to estimate R_0_ for a given prevalence value [6]. This model assumes various density-dependent functions which alter the rates of transition between state variables in the model (for example, the rate of parasite establishment and rate of parasite fecundity). The model parameter prior distributions were defined using data from the literature and parameter posteriors were found by fitting to baseline mf prevalence data from low, moderate and high transmission settings in Tanzania [52] – settings which are representative of much of SSA.

For a setting with a particular endemic prevalence, there is an underlying bite rate and force of infection which leads to this endemic equilibrium. This parameter contributes to R_0_ (as explained in detail below), but can only be calculated indirectly through the method described in detail by Gambhir et al. [53] and outlined here.

The first stage in estimating R_0_ is to calculate the effective reproduction number, R_eff_ in terms of the constant terms in the mathematical model and the density-dependent functions of the state variables. The effective reproductive number, R_eff_, is equal to the basic reproductive number when a parasite is newly introduced to a population, but as parasites become established in the population, density dependent effects, such a density dependent fecundity, can both limit and facilitate transmission, increasing or decreasing R_eff_. When the system is at equilibrium, the effective reproductive number is one (the number of parasites is neither increasing nor decreasing) and the parasite load in the population is at its equilibrium value. Therefore, we want to calculate the value of the biting rate parameters for which this expression equals 1 for a particular population parasite load. The expression for R_eff_ is an implicit expression which cannot be solved analytically, and therefore has to be solved numerically for particular settings using the following method. When the function R_eff_ is plotted against a population state variable (such as mf prevalence or community mf load), the resulting plot increases with parasite density in the population for low levels of parasite load, but for large levels of parasite load in the host population density dependent processes decrease R_eff_, leading to a “humped” function (Fig. 1 of Gambhir et al. [53]), i.e. as mf load (or prevalence) is varied, the function rises or falls corresponding to increasing or decreasing LF transmission. This humped function has a maximum. When this peak value of R_eff_ is more than 1, the effective reproduction number intersects the R_eff_ = 1 line twice, meaning that there are two equilibria [53]. The higher of these is the stable endemic equilibrium, and the lower is an unstable extinction ‘breakpoint’ [54]. Therefore in the absence of treatment, we assume that the higher point represents LF prevalence at stable endemic equilibrium (i.e. pre-intervention). Specifically, in order to fit the R_eff_ function to the mf prevalence data, so that its upper equilibrium corresponds to the observed endemic prevalence value, we alter the mosquito-human biting rate parameter – the rate at which humans are bitten by mosquitoes – until this correspondence occurs. The model parameter values denoting a given endemic prevalence value, are used in Gambhir et al., equation 6 [53] to estimate the value of *R*_*0*_:

$$ {R}_0=\frac{\lambda {f}_1(0){f}_2(0)\alpha {f}_3(0)\beta {f}_4(0)}{\mu \delta \sigma } $$

where *λ*, *α* and *β* are the ‘immigration’ rates of each of the life stages (adult worms, microfilariae, and L3 infective larvae respectively; *λ* is a composite parameter, incorporating the mosquito-human biting rate which is estimated using the methodology describe above; *μ*, *δ* and *σ* are the death rates of each of the life stages respectively; and *f*_1_(*I*), *f*_2_(*W*), *f*_3_(*W*), *f*_4_(*M*) represent the modifying density-dependent functions acting on each of the respective parasite life-stage intensities (*W*, the number of adult worms per definitive host, *M*, the mf load per host, refer to parasite life stages within the definitive host population, whereas the L3 infective larval load per mosquito (larvae develop through L1 and L2 stages but only become infective once they reach the L3 stage), *L*, refers to the parasite life-stage within the vector host. The host immunity variable, *I*, increases in magnitude at a rate equal to the adult worm burden but can also decay over time, allowing the model to capture the waning of immunity). Note that for *R*_*0*,_ host immunity levels and worm burdens are assumed to be zero as this expression calculates the number of offspring in a wholly susceptible population. The above mathematical model was initially fitted separately for settings where either *Anopheles* or *Culex* species are the predominant vector, based on the develop vector distribution maps (Fig. 3). However, the relationship between the mf prevalence and R_0_ is practically identical for *Anopheles* or *Culex* species, as the model assumes similar mosquito life expectancy for each species, and therefore we present a single relationship between prevalence of mf and R_0_. Finally, the threshold mf prevalence value above which LF transmission can persist, which is higher than the prevalence at which *R*_0_ = 1 due to the density dependencies assumed in the modelling framework [53], was calculated.

## Results

### Collated survey data

The literature search identified 1224 surveys that provide data on the prevalence of mf and 3519 surveys that provide suitable data on the prevalence of antigenaemia, as estimated by using an ICT (Fig. 2). Most of the mf data arose from surveys conducted between 1950 and1969 or from 1990 onwards, whereas the ICT data were mainly collected from 2000 onwards (Table 1). Figure 2 maps the spatial distribution of the included mf and ICT data and shows that the majority of data were available from east and west Africa.

**Table 1:** Summary of data on the prevalence of microfilaraemia (mf) and prevalence of antigenaemia, based on immuno-chromatographic card test (ICT) by region and time period. Median and inter-quartile range (IQR) are presented

	Mf prevalence						ICT prevalence	
	1950–1969		1970–1989		1990–onwards		1990–onwards	
Region	N	Median (IQR)	N	Median (IQR)	N	Median (IQR)	N	Median (IQR)
Eastern	46	14.1 (2.6–34.6)	109	15.8 (4.6–30.2)	137	11.5 (2.8–21.6)	2002	0 (0–1)
Middle	21	0 (0–2.7)	38	0 (0–0)	31	1 (0–1.2)	564	1 (0–5.5)
Northern	3	22 (0–26.8)	1	10	-	-	-	-
Western	304	6.8 (1.2–21.7)	190	7.3 (2–15.9)	265	6.3 (1.2–15.1)	629	6 (0–26)
Total	374	7.1 (1.1–22)	338	8.1 (0.8–18.9)	433	6.5 (1.2–17)	3195	0 (0–4.8)

### Vector distribution maps

Figure 3 presents maps of the predicted distributions of *Anopheles, Culex,* and *Mansonia* species. *Anopheles* mosquitoes of *gambiae* and *funestus* complexes are widely distributed across sub-Saharan Africa (Fig. 3a). In contrast, *M. africana* and *Culex* mosquitoes show a more limited and distinct distribution: *Culex* mosquitoes occur in eastern Africa, east coast of Madagascar and in restricted areas of west Africa (Fig. 3b), whereas *M. africana* occurs in west and middle Africa, and coastal areas of east Africa and Madagascar (Fig. 3c). A wider presence of *Culex* mosquitoes is predicted in Nigeria, north-east of Cameroon and north of Angola. Areas where the distribution of each of the major LF mosquito vectors occur across west Africa and coastal areas of east Africa (Fig. 3d).

### Spatial prediction of LF prevalence

**Fig. 4:**
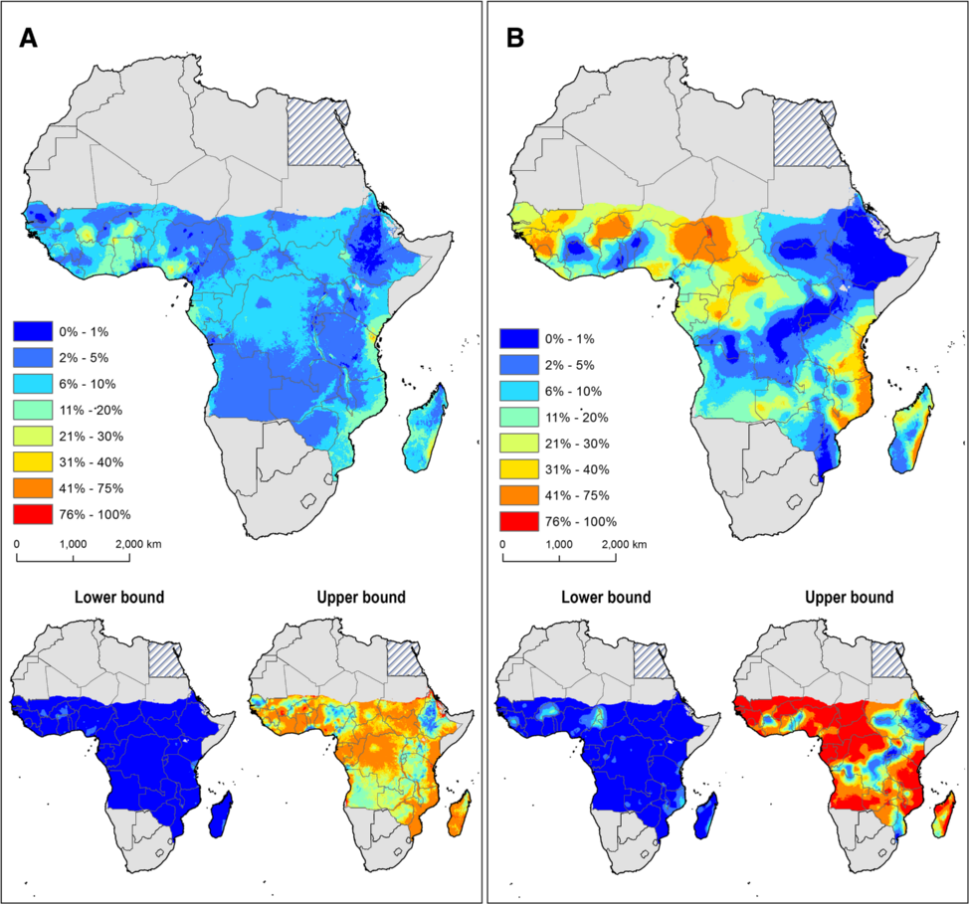
Predicted geographical distribution of the prevalence of **a** microfilaraemia and **b** antigenaemia, based on a Bayesian geostatistical modelling approach for the period 1990–onwards and before the implementation of large-scale interventions. Point estimates (based on posterior median) together with lower (2.5 %) and upper (97.5 %) percentiles are presented

In the spatial model for ICT prevalence, the model selection process excluded land surface temperature, slope and distance to water bodies, while in the spatiotemporal model for mf prevalence no covariates were excluded during variable selection, as based on the AIC criterion (Additional file 2: Table S5). The predicted prevalence of LF, based on mf and ICT-based data, are presented in Fig. 4a and b, respectively, along with estimates of 2.5 and 97.5 % quantiles. Overall, predicted mf prevalence is lower than predicted ICT prevalence across sub-Saharan Africa, not exceeding the 5 % threshold in most endemic areas. Only a few small pockets of mf prevalence higher than 30–40 % are predicted in east Africa, at the north of coastal Tanzania and the southeast coast of Madagascar. Broader areas of high mf prevalence are predicted in west Africa; south of Mali (Sikasso region), large central areas of Benin and north-west of Ghana (bordering with Benin) and at the south of Abuja, the federal capital of Nigeria.

The ICT-based model indicate large areas of high prevalence (above 40 %) are predicted along the coast of east Africa, from southern Kenya to central regions of Mozambique, and the east coast of Madagascar. High prevalences of antigenaemia are predicted across large areas of west Africa and pockets at the north-west of the Democratic Republic of Congo (DRC) and central Congo. In general, lower (<5 %) estimates of ICT prevalence are predicted in inland areas of east Africa (Ethiopia, Rwanda, Burundi, central and south of Uganda) and middle Africa (southern and central DRC).

Validation of the spatiotemporal model for mf prevalence was based on 86 locations and yielded a correlation coefficient between predicted and observed values of 0.69 and a mean error of 1.89 % - this indicates that, on average, the model predictions overestimate the observed prevalence by 1.89 %. The mean absolute error, which illustrates the average magnitude of the prediction errors, was 4.64 % and the percentage of locations from the sample with observed prevalence falling in the 95 % CI was 70.93 % (Table 2). Validation of the spatial model for ICT-based prevalence was based on 639 locations and revealed a correlation coefficient of 0.86, mean error of 0.06 %, and mean absolute error of 6.51 %. The percentage of locations from the sample with observed prevalence falling in the 95 % CI was 52.83 % - this low percentage was due to the fact that intervals do not cover the actual values which are observed in locations where prevalence is 0. The coverage percentage increased to 77.49 % if we approximated by 0 the interval lower limits which are lower than 0.01 %.

**Table 2:** Validation statistics for spatio-temporal model for mf prevalence and spatial model for ICT-based prevalence

Model	Correlation	Mean Error	Mean Absolute Error	Coverage percentage of 95 % CI
mf	0.69	1.89 %	4.64 %	70.93 %
ICT	0.86	0.06 %	6.51 %	52.83 %^a^, 77.49 %^b^

For the ICT data, coverage percentages are presented for the actual intervals^a^ and for intervals where lower limits turned out lower than 0.01 % those were replaced by 0^b^

### Predictive distribution of R_0_

**Fig. 5:**
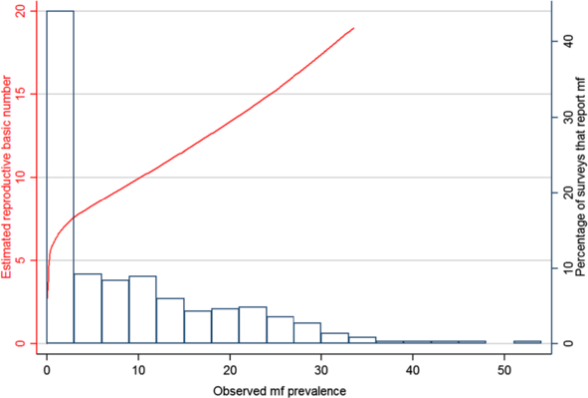
The modelled relationship between prevalence of microfilaraemia (mf) and the reproduction number (R_0_). Also shown is the distribution of observed mf prevalence (1990–onwards) included in the mapping analysis (*n* = 434 surveys)

**Fig. 6:**
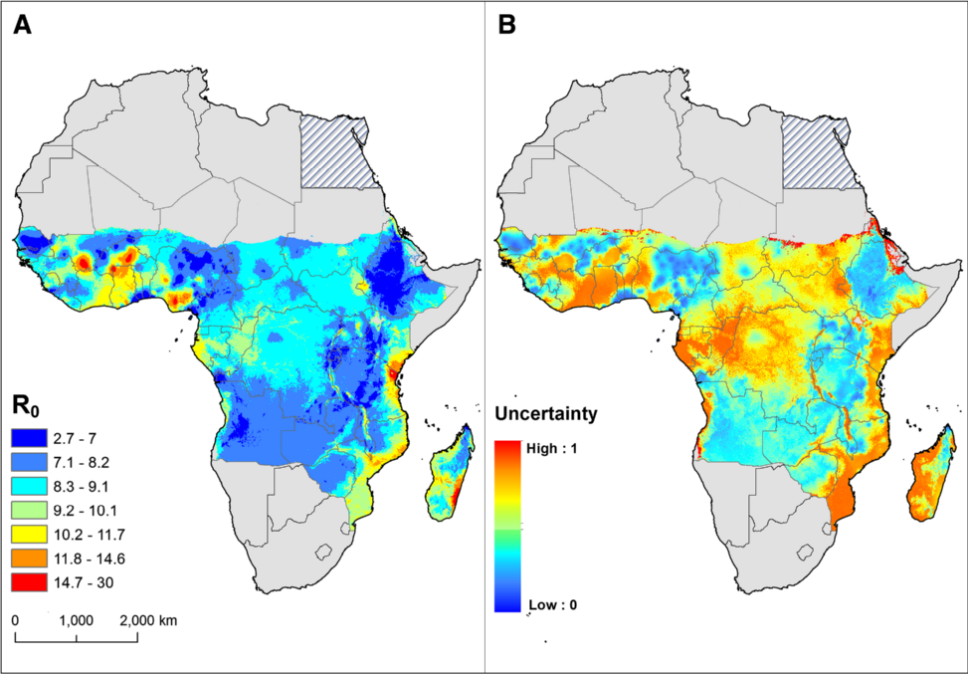
Geographical distribution of lymphatic filariasis basic reproductive number (**a**) and uncertainty on R_0_ estimates (**b**) prior to the large-scale implementation of interventions. Uncertainty was calculated as the range of the 95 % confidence interval in predicted R_0_ estimates for each pixel and rescaling to a 0–1 scale

The relationship between the prevalence of mf and R_0_ is presented in Fig. 5. Figure 6 shows a predicted map of R_0_ based on mf prevalence. The estimated R_0_ values vary from 2.7 to 30 across sub-Saharan Africa. The areas of low and moderate intensity of LF transmission (R_0_ value lower than 10) predominate in all endemic areas and the highest intensity is predicted in restricted areas of west Africa (Nigeria, Ghana and Burkina Faso), along the coast of east Africa, from north-east of Kenya to southern Mozambique and larger areas in central and eastern Madagascar.

## Discussion

Here we present maps of the distribution of LF microfilaraemia and antigenaemia across SSA under a pre-intervention scenario, based on Bayesian geostatistical modelling. We also present the first attempt to map geographical variation in R_0_, which reflects differential risks of recrudescence and can be used to inform the design of post-MDA surveillance activities.

The geostatistical maps presented here indicate lower and more focal patterns of mf compared patterns of antigenaemia. The maps also suggest a more restricted distribution of mf compared to previous mapping work by Slater and Michael [11] which suggested higher levels of infection overall and the occurrence of transmission where LF has never or only occasionally been reported. For example, the Slater and Michael model predict mf prevalences above 75 % in Sudan and South Sudan-Ethiopia border, whereas our predicted prevalence <10 % and recent surveys using ICTs found low levels of antigenaemia [55–57]. Our predicted distribution of antigenaemia is also consistent with previous country-level models [8, 58–60]. Uncertainty in predictions based on mf prevalence data is greatest for central Africa, primarily driven by a scarcity of mf surveys conducted since 1990. This lack of contemporary data is compensated in part by the inclusion of older data and a temporal effect in the final model.

Here we also present the first composite map of the distribution of dominant LF vector species in SSA. *Anopheles* mosquitoes, mostly those species belonging to the *gambiae* and *funestus* complexes, are considered the major LF vector in SSA, particularly in rural settings where LF is more prevalent [33]. Mosquitoes of the *Cx. pipiens* complex, especially *Cx. quinquefasciatus,* are suggested to be important vector of LF in eastern Africa, especially in urban and peri-urban settings [61, 62]. In west Africa, however, it is widely accepted that *Anopheles* species are the main vectors since *Culex* mosquitoes, despite being ubiquitous and potential vectors in the region [63], are considered to transmit LF poorly [64–66]. Interestingly, the areas where at least two vector genera occur coincide with areas showing the highest estimates of antigenaemia and microfilaraemia. This apparent concordance makes us think that a more efficient and higher intensity of LF transmission is expected where multiple potential LF vectors occur.

### Strengths and challenges in LF mapping

The presented work has a number of strengths and limitations. Our use of Bayesian model-based geostatistics provides a flexible and robust approach for spatial modelling that takes into account the spatial dependence structure of the data and provides a formal expression of uncertainty in the prediction estimates [67]. Traditionally, Bayesian predictive inference has been implemented via MCMC methods which make inference tractable for complex models but present problems in terms of convergence and computational time [68]. We overcome these issues by using the INLA approach [46] which is a computationally effective alternative to MCMC designed for latent Gaussian models. By using a combination of analytical approximation and numerical integration, INLA produces fast and accurate approximations to posterior distributions. The analysis of spatial point data is possible by combining INLA and SPDE approaches [47], whereby a continuously indexed Gaussian field is represented as a discretely indexed Gaussian Markov random field (GMRF) using a basis function representation defined on a triangulation of the domain of interest.

Intensity of LF transmission is influenced by a complexity of factors, yet our models incorporated only environmental and demographic drivers of LF transmission in pre-intervention scenarios. The models do not incorporate socioeconomic status [69–72] or the coverage of LF interventions, including MDA and vector control, which will influence transmission, especially once interventions have been scaled up. The role of vector control measures in modifying patterns of LF transmission has recently been highlighted in The Gambia where the large-scale distribution of bed nets for malaria control has, in part, contributed to the elimination of LF in the country [73]. In coastal Kenya, large-scale distribution of ITNs for malaria control has been proposed to sustain reduction of LF infection levels, even after MDA is interrupted [74]. An increasing number of studies have demonstrated the impact of repeated rounds of albendazole and ivermectin alter patterns of transmission [75] and over time dramatically reduce transmission [76, 77]. A methodological challenge for incorporating intervention-related factors into future models is that much of the available data are presented at an area-level. Bayesian hierarchical models have been used to address this problem by providing a natural way to combine data from different sources taking into account the different uncertainties [78–80]. However, such models have three important limitations. First, they rely on MCMC methods for Bayesian inference which are computationally intensive and may become unfeasible for large datasets. Second, few approaches for temporally misaligned data exist and they do not account for correlation in spatial random effects over time. Third, correlation studies use the predictions from several models as covariates in regression models and as such predictions contain error as the predicted values do not equal the true exposures. These important issues indicate that there is considerable scope to develop new model-based methods for the analysis of misaligned data.

The vector distribution maps are also not without their limitations. First, different methodologies were used for different genera: maps for *Culex* and *Mansonia* were constructed by using maximum entropy ecological niche (MaxEnt) modelling [40]; and boosted regression tree (BRT) modelling was used to modelling environmental suitability for *Anopheles* mosquitoes [38]. Second, there is a lack of data for some areas of central and northeast Africa, introducing imprecision into maps.

Finally, the presented map of R0 is based on a mathematical model of transmission dynamics [53]. The relationship between prevalence and R0 is non-linear and there are very high values of R0 for moderate to high levels of prevalence, suggesting great challenges for control. However, it should be remembered that these values are for a population when there is no immunity, unlike endemic communities, and that we do not yet have a reliable estimate for how long this immunity will last. Therefore the high values of R0 may not represent such a large challenge for control in the short term. For recrudescence or re-emergence however, they may represent a higher risk. The model uses prevalence to estimate transmission intensity because of the availability of prevalence data. Alternative measures of transmission intensity include annual transmission potential and annual biting rates [81], but these are very rarely measured. Ideally, the model would have been parameterised with data from a range of transmission settings, but there are a lack of detailed data and we recognise that transmission dynamics will vary in settings other than Tanzania from where data were used to parameterise the model.

### Mapping LF to guide control and surveillance

The developed map of R_0_ can be used to identify areas of higher transmission intensity where additional control efforts may be required to achieve the elimination of LF infection (i.e. implementing vector control or extending MDA rounds). Our model predicts a marked geographical heterogeneity in the intensity of transmission in SSA. Current MDA strategies do not account for this spatial heterogeneity, and therefore coverage targets and implementation schemes are common for all endemic areas [3]. The precise level and duration of treatments to achieve LF elimination in different endemic regions remains unknown [82], such that it is difficult to decide when to stop ongoing MDA interventions [51]. Indeed, although some model predictions suggest that LF transmission can be interrupted by annual MDA alone, it is not clear that this can be achieved everywhere with 4–6 yearly rounds of MDA [83].

In recent years, some experts have suggested that current control programmes based on standard MDA schemes should give rise to more tailored control interventions adapted to the local specificities of LF transmission [6, 14, 84]. Our maps provide programme managers with a thorough picture of the initial level of LF endemicity and potential geographical variation in the intensity of transmission prior to the implementation of large-scale interventions. The R_0_ maps also provide insight into (i) the risk of persistent transmission despite repeated MDA and highlight that pockets of residual transmission may occur in settings where overall transmission has declined [77], and (ii) the possibility of recrudescence, especially where transmission is highly heterogeneous [85]. Careful use of the developed maps can guide decision makers and ultimately programme managers first to define the most appropriate control strategy and adapt it to local conditions of transmission and second to identify areas which may require special attention during monitoring and surveillance stages. How to integrate an understanding of the spatial heterogeneity of LF into the design of optimal LF surveillance is the subject of ongoing work.

## Conclusions

The transmission of LF is spatially heterogeneous and it is essential that intervention, monitoring and surveillance strategies take this variation into account. The developed maps of mf, antigenaemia and R_0_ are intended to contribute to this effort. In particular, our maps can help guide control programmes in future LF surveillance activities by identifying areas of higher intensity of transmission that may require enhanced and tailored interventions and by setting a benchmark for evaluating the impact of scaling up of LF interventions on the pathway to LF elimination. Country maps displaying the results of this modelling work will be available at the website of the Global Atlas of Helminth Infection project (www.thiswormyworld.org).

## Additional files

Additional file 1:
**Characteristics of surveys used to develop the mf and ICT models.** Standardization of mf prevalence based on blood volume. **Table S1**. Blood samples and diagnostic methods of surveys included in the global LF database (African region). **Table S2**. Paired estimates of LF prevalence based on blood smear and filtration methods. (DOCX 30 kb) Additional file 2:
**Environmental and demographic covariates used in the geostatistical modelling.** **Table S3**. Description of the covariates explored to model the microfilaraemia and antigenaemia prevalence. **Table S4**. Correlation matrix of environmental and demographic covariates (continuous data) included in the modelling. Spearman’s correlation test was used to explore the colinearity between pairs of covariates. **Table S5**. Relationship between infection risk and each potential explanatory variable. (DOCX 34 kb)

## References

[1] Ramaiah KD, Ottesen EA (2014). Progress and impact of 13 years of the global programme to eliminate lymphatic filariasis on reducing the burden of filarial disease. PLoS Negl Trop Dis.

[2] WHO (2013). Report of an informal meeting on transmission assessment surveys for review of the training modules and coordination for country support.

[3] WHO (2011). Monitoring and Epidemiological Assessment of Mass Drug Administration for the Global Programme to Eliminate Lymphatic Filariasis (GPELF).

[4] Garrett-Jones C (1964). Prognosis for Interruption of Malaria Transmission through Assessment of the Mosquito’s Vectorial Capacity. Nature.

[5] Gambhir M, Michael E (2008). Complex ecological dynamics and eradicability of the vector borne macroparasitic disease, lymphatic filariasis. PLoS One.

[6] Gambhir M, Bockarie M, Tisch D, Kazura J, Remais J, Spear R (2010). Geographic and ecologic heterogeneity in elimination thresholds for the major vector-borne helminthic disease, lymphatic filariasis. BMC Biol.

[7] Stanton MC, Mkwanda S, Mzilahowa T, Bockarie MJ, Kelly-Hope LA (2014). Quantifying filariasis and malaria control activities in relation to lymphatic filariasis elimination: a multiple intervention score map (MISM) for Malawi. Trop Med Int Health.

[8] Stanton MC, Molyneux DH, Kyelem D, Bougma RW, Koudou BG, Kelly-Hope LA (2013). Baseline drivers of lymphatic filariasis in Burkina Faso. Geospat Health.

[9] Kelly-Hope LA, Thomas BC, Bockarie MJ, Molyneux DH (2011). Lymphatic filariasis in the Democratic Republic of Congo; micro-stratification overlap mapping (MOM) as a prerequisite for control and surveillance. Parasit Vectors.

[10] Stensgaard AS, Vounatsou P, Onapa AW, Simonsen PE, Pedersen EM, Rahbek C (2011). Bayesian geostatistical modelling of malaria and lymphatic filariasis infections in Uganda: predictors of risk and geographical patterns of co-endemicity. Malar J.

[11] Slater H, Michael E (2013). Mapping, Bayesian Geostatistical Analysis and Spatial Prediction of Lymphatic Filariasis Prevalence in Africa. PLoS One.

[12] Cano J, Rebollo MP, Golding N, Pullan RL, Crellen T, Soler A (2014). The global distribution and transmission limits of lymphatic filariasis: past and present. Parasit Vectors.

[13] Brooker S, Hotez PJ, Bundy DA (2010). The global atlas of helminth infection: mapping the way forward in neglected tropical disease control. PLoS Negl Trop Dis.

[14] Weil GJ, Ramzy RM (2007). Diagnostic tools for filariasis elimination programs. Trends Parasitol.

[15] Rebollo MP, Bockarie MJ (2014). Shrinking the lymphatic filariasis map: update on diagnostic tools for mapping and transmission monitoring. Parasitology.

[16] Michael E, Malecela MN, Zervos M, Kazura JW (2008). Global eradication of lymphatic filariasis: the value of chronic disease control in parasite elimination programmes. PLoS One.

[17] Sabry M (1991). A quantitative approach to the relationship between Wuchereria bancrofti microfilaria counts by venous blood filtration and finger-prick blood films. Trans R Soc Trop Med Hyg.

[18] Weil GJ, Lammie PJ, Weiss N (1997). The ICT Filariasis Test: A rapid-format antigen test for diagnosis of bancroftian filariasis. Parasitol Today.

[19] Bakajika DK, Nigo MM, Lotsima JP, Masikini GA, Fischer K, Lloyd MM (2014). Filarial Antigenemia and Loa loa Night Blood Microfilaremia in an Area Without Bancroftian Filariasis in the Democratic Republic of Congo. Am J Trop Med Hyg.

[20] Zoure HG, Wanji S, Noma M, Amazigo UV, Diggle PJ, Tekle AH (2011). The geographic distribution of Loa loa in Africa: results of large-scale implementation of the Rapid Assessment Procedure for Loiasis (RAPLOA). PLoS Negl Trop Dis.

[21] WHO (2014). Global programme to eliminate lymphatic filariasis: progress report, 2013. Wkly Epidemiol Rec.

[22] WHO (2013). Sustaining the drive to overcome the global impact of neglected tropical diseases: second WHO report on neglected diseases.

[23] Brooker S, Michael E (2000). The potential of geographical information systems and remote sensing in the epidemiology and control of human helminth infections. Adv Parasitol.

[24] Lindsay SW, Thomas CJ (2000). Mapping and estimating the population at risk from lymphatic filariasis in Africa. Trans R Soc Trop Med Hyg.

[25] Slater H, Michael E (2012). Predicting the current and future potential distributions of lymphatic filariasis in Africa using maximum entropy ecological niche modelling. PLoS One.

[26] WorldClim. Global Climate data. http://www.worldclim.org/.

[27] AfSIS. Africa Soil Information Service. ftp://africagrids.net/.

[28] CGIAR-CSI. Consortium for Spatial Information. http://www.cgiar-csi.org/.

[29] JRS. JRS-Land Resource Management Unit. Global Land Cover 2000. https://ec.europa.eu/jrc/en/scientific-tool/global-land-cover.

[30] WorldPop. The AfriPop demography project. http://www.worldpop.org.uk/.

[31] UNEP/GRID. UNEP/GRID-Sioux Falls. African Population Distribution Database. 2000 Population Density. http://na.unep.net/siouxfalls/datasets/datalist.php.

[32] de Smith MJ, Goodchild MF, Longley PA (2007). Geospatial Analysis. A comprehensive guide to principles. techniques and software tools.

[33] Bockarie MJ, Pedersen EM, White GB, Michael E (2009). Role of vector control in the global program to eliminate lymphatic filariasis. Annu Rev Entomol.

[34] Rebollo MP, Bockarie MJ (2013). Toward the elimination of lymphatic filariasis by 2020: treatment update and impact assessment for the endgame. Expert Rev Anti Infect Ther.

[35] Pichon G (2002). Limitation and facilitation in the vectors and other aspects of the dynamics of filarial transmission: the need for vector control against Anopheles transmitted filariasis. Ann Trop Med Parasitol.

[36] Michael E, Snow LC, Bockarie MJ (2009). Ecological meta-analysis of density-dependent processes in the transmission of lymphatic filariasis: survival of infected vectors. J Med Entomol.

[37] Bockarie M, Pedersen E, White G, Michael E (2009). Role of Vector Control in the Global Program to Eliminate Lymphatic Filariasis. Ann Rev Entomol.

[38] Sinka ME, Bangs MJ, Manguin S, Rubio-Palis Y, Chareonviriyaphap T, Coetzee M (2012). A global map of dominant malaria vectors. Parasit Vectors.

[39] Sinka ME, Bangs MJ, Manguin S, Coetzee M, Mbogo CM, Hemingway J (2010). The dominant Anopheles vectors of human malaria in Africa, Europe and the Middle East: occurrence data, distribution maps and bionomic precis. Parasit Vectors.

[40] VectorMap. The Mosquito Map project. http://www.mosquitomap.org/.

[41] Stevens KB, Pfeiffer DU (2011). Spatial modelling of disease using data- and knowledge-driven approaches. Spat Spatiotemporal Epidemiol.

[42] GBIF. Global Biodiversity Information Facility. http://www.gbif.org/.

[43] Cano J, Moraga P, Nikolay B, Rebollo MP, Okorie PN, Davies E (2015). An investigation of the disparity in estimates of microfilaraemia and antigenaemia in lymphatic filariasis surveys. Trans R Soc Trop Med Hyg.

[44] Pullan RL, Gething PW, Smith JL, Mwandawiro CS, Sturrock HJ, Gitonga CW (2011). Spatial modelling of soil-transmitted helminth infections in Kenya: a disease control planning tool. PLoS Negl Trop Dis.

[45] Rue H, Martino S (2007). Approximate Bayesian inference for hierarchical Gaussian Markov random field models. J Stat Plan Inference.

[46] Rue H, Martino S, Chopin N (2009). Approximate Bayesian inference for latent Gaussian models by using integrated nested Laplace approximations. Appl Stat.

[47] Lindgren F, Rue H, Lindström J (2011). An explicit link between Gaussian fields and Gaussian Markov random fields: the stochastic partial differential equation approach. Appl Stat.

[48] Anderson RM, May RM (1992). Infectious diseases of humans. Dynamics and control.

[49] Basanez MG, Collins RC, Porter CH, Little MP, Brandling-Bennett D (2002). Transmission intensity and the patterns of Onchocerca volvulus infection in human communities. Am J Trop Med Hyg.

[50] Dietz K. The population dynamics of onchocerciasis. In: The Population Dynamics of Infectious Diseases: Theory and Applications. Ed RM Anderson. ISBN: 978-0-412-21610-7 (Print) 978-1-4899-2901-3 (Online) p209-241. 1982.

[51] Michael E, Malecela-Lazaro MN, Kabali C, Snow LC, Kazura JW (2006). Mathematical models and lymphatic filariasis control: endpoints and optimal interventions. Trends Parasitol.

[52] Michael E, Simonsen PE, Malecela M, Jaoko WG, Pedersen EM, Mukoko D (2001). Transmission intensity and the immunoepidemiology of bancroftian filariasis in East Africa. Parasite Immunol.

[53] Gambhir M, Singh BK, Michael E (2015). The Allee effect and elimination of Neglected Tropical Diseases: A mathematical modelling study. Adv Parasitol.

[54] May RM (1977). Thresholds and breakpoints in ecosystems with a multiplicity of stable states. Nature.

[55] Shiferaw W, Kebede T, Graves PM, Golasa L, Gebre T, Mosher AW (2012). Lymphatic filariasis in western Ethiopia with special emphasis on prevalence of Wuchereria bancrofti antigenaemia in and around onchocerciasis endemic areas. Trans R Soc Trop Med Hyg.

[56] Finn TP, Stewart BT, Reid HL, Petty N, Sabasio A, Oguttu D (2012). Integrated rapid mapping of neglected tropical diseases in three States of South Sudan: survey findings and treatment needs. PLoS One.

[57] Sturrock HJ, Picon D, Sabasio A, Oguttu D, Robinson E, Lado M (2009). Integrated mapping of neglected tropical diseases: epidemiological findings and control implications for northern Bahr-el-Ghazal State. Southern Sudan PLoS Negl Trop Dis.

[58] Mwase ET, Stensgaard A-S, Nsakashalo-Senkwe M, Mubila L, Mwansa J, Songolo P (2014). Mapping the Geographical Distribution of Lymphatic Filariasis in Zambia. PLoS Negl Trop Dis.

[59] Koroma JB, Bangura MM, Hodges MH, Bah MS, Zhang Y, Bockarie MJ (2012). Lymphatic filariasis mapping by immunochromatographic test cards and baseline microfilaria survey prior to mass drug administration in Sierra Leone. Parasit Vectors.

[60] Gyapong JO, Kyelem D, Kleinschmidt I, Agbo K, Ahouandogbo F, Gaba J (2002). The use of spatial analysis in mapping the distribution of bancroftian filariasis in four West African countries. Ann Trop Med Parasitol.

[61] Pedersen EM, Kilama WL, Swai AB, Kihamia CM, Rwiza H, Kisumku UM (1999). Bancroftian filariasis on Pemba Island, Zanzibar, Tanzania: an update on the status in urban and semi-urban communities. Trop Med Int Health.

[62] Maxwell CA, Curtis CF, Haji H, Kisumku S, Thalib AI, Yahya SA (1990). Control of Bancroftian filariasis by integrating therapy with vector control using polystyrene beads in wet pit latrines. Trans R Soc Trop Med Hyg.

[63] Knight KL, Stone A (1977). A Catalog of the Mosquitoes of the World (Diptera: Culicidae).

[64] Subra R, Mouchet J. Culex pipens fatigans Wiedemann in West Africa and its possible role in the transmission of Bancroft’s filariasis 1967 Contract No.: WHO/FIL/67.73.

[65] Zielke E, Kuhlow F (1977). On the inheritance of susceptibility for infection with Wuchereria bancrofti in Culex pipiens fatigans. Tropenmed Parasitol.

[66] Jayasekera N, Curtis CF, Zielke E, Kuhlow F, Jansen CG, Chelliah RV (1980). The susceptibility of Liberian Culex quinquefasciatus to Wuchereria bancrofti in Sri Lanka. Tropenmed Parasitol.

[67] Diggle PD, Ribeiro PJ (2007). Model-based geostatistics.

[68] Taylor BT, Diggle PD (2014). INLA or MCMC? A tutorial and comparative evaluation for spatial prediction in log-Gaussian Cox processes. J Stat Comput Simul.

[69] Bonfim C, Alves A, Costa TR, Alencar F, Pedroza D, Portugal JL (2011). Spatial analysis and privation index to identify urban areas with a high risk of lymphatic filariasis. Trop Med Int Health.

[70] Upadhyayula SM, Mutheneni SR, Kadiri MR, Kumaraswamy S, Nagalla B (2012). A cohort study of lymphatic filariasis on socio economic conditions in Andhra Pradesh. India PLoS One.

[71] Tyrell E (2013). Socioeconomic burden of lymphatic filariasis in Georgetown, Guyana. Trop Med Int Health.

[72] Simonsen PE, Mwakitalu ME (2013). Urban lymphatic filariasis. Parasitol Res.

[73] Rebollo MP, Sambou SM, Thomas B, Biritwum NK, Jaye MC, Kelly-Hope L, et al. Elimination of lymphatic filariasis in The Gambia. PLoS Negl Trop Dis. 2015;9(3):e0003642. doi:10.1371/journal.pntd.0003642. 10.1371/journal.pntd.0003642 PMC4364952 25785587

[74] Njenga SM, Mwandawiro CS, Wamae CN, Mukoko DA, Omar AA, Shimada M, et al. Sustained reduction in prevalence of lymphatic filariasis infection in spite of missed rounds of mass drug administration in an area under mosquito nets for malaria control. Parasit Vectors. 2011;4:90. 10.1186/1756-3305-4-90 PMC3125382 21612649

[75] Coulibaly YI, Dembele B, Diallo AA, Kristensen S, Konate S, Dolo H (2013). Wuchereria bancrofti transmission pattern in southern Mali prior to and following the institution of mass drug administration. Parasit Vectors.

[76] Richards FO, Eigege A, Miri ES, Kal A, Umaru J, Pam D (2011). Epidemiological and entomological evaluations after six years or more of mass drug administration for lymphatic filariasis elimination in Nigeria. PLoS Negl Trop Dis.

[77] Coulibaly YI, Dembele B, Diallo AA, Konate S, Dolo H, Coulibaly SY (2015). The Impact of Six Annual Rounds of Mass Drug Administration on Wuchereria bancrofti Infections in Humans and in Mosquitoes in Mali. Am J Trop Med Hyg.

[78] Mugglin AS, Carlin BP, Gelfand AE (2000). Fully Model-Based Approaches for Spatially Misaligned Data. J Am Stat Assoc.

[79] Gelfand AE, Zhu L, Carlin BP (2001). On the change of support problem for spatio-temporal data. Biostatistics.

[80] Fuentes M, Raftery AE (2005). Model evaluation and spatial interpolation by Bayesian combination of observations with outputs from numerical models. Biometrics.

[81] Kazura JW, Bockarie M, Alexander N, Perry R, Bockarie F, Dagoro H (1997). Transmission intensity and its relationship to infection and disease due to Wuchereria bancrofti in Papua New Guinea. J Infect Dis.

[82] Michael E, Malecela-Lazaro MN, Maegga BT, Fischer P, Kazura JW (2006). Mathematical models and lymphatic filariasis control: monitoring and evaluating interventions. Trends Parasitol.

[83] Stolk WA, Stone C, de Vlas SJ (2015). Modelling lymphatic filariasis transmission and control: modelling frameworks, lessons learned and future directions. Adv Parasitol.

[84] Okorie PN, Ademowo GO, Saka Y, Davies E, Okoronkwo C, Bockarie MJ (2013). Lymphatic Filariasis in Nigeria; Micro-stratification Overlap Mapping (MOM) as a Prerequisite for Cost-Effective Resource Utilization in Control and Surveillance. PLoS Negl Trop Dis.

[85] Rebollo MP, Mohammed KA, Thomas B, Ame S, Ali SM, Cano J (2015). Cessation of mass drug administration for lymphatic filariasis in Zanzibar in 2006: was transmission interrupted?. PLoS Negl Trop Dis.

