# Supplementary material for: Modelling the distribution and transmission intensity of lymphatic filariasis in sub-Saharan Africa prior to scaling up interventions: integrated use of geostatistical and mathematical modelling
Source: Parasit Vectors. 2015 Oct 24;8:560. doi: 10.1186/s13071-015-1166-x (PMC4620019; doi:10.1186/s13071-015-1166-x)
Supplement: Additional file 1: — Characteristics of surveys used to develop the mf and ICT models. Standardization of mf prevalence based on blood volume. Table S1. Blood samples and diagnostic methods of surveys included in the global LF database (African region). Table S2. Paired estimates of LF prevalence based on blood smear and filtration methods. (DOCX 30 kb) [file 13071_2015_1166_MOESM1_ESM.docx]

**Additional file 1**

**Characteristics of surveys used to develop the mf and ICT models**

| **Characteristics** |  | **ICT surveys** | | **Mf surveys** | | **Total** | |
| --- | --- | --- | --- | --- | --- | --- | --- |
|  |  | **N** | **%** | **N** | **%** | **N** | **%** |
| **Total** |  | **3,195** |  | **1,224** |  | **4,419** |  |
| Surveys by sub-region | |  | |  | |  | |
|  | *Eastern Africa* | 2,002 | 62.7 | 300 | 24.5 | 2,302 | 52.1 |
|  | *Middle Africa* | 564 | 17.7 | 138 | 11.3 | 702 | 15.9 |
|  | *Northern Africa* | 0 | 0.0 | 4 | 0.3 | 4 | 0.1 |
|  | *Western Africa* | 629 | 19.7 | 782 | 63.9 | 1,411 | 31.9 |
| Surveys by period | |  |  |  |  |  |  |
|  | *before 1990* | 0 | 0.0 | 791 | 64.6 | 791 | 17.9 |
|  | *1990-2000* | 17 | 0.5 | 115 | 9.4 | 132 | 3.0 |
|  | *2000-now* | 3,178 | 99.5 | 318 | 26.0 | 3,496 | 79.1 |
| Source of surveys | |  |  |  |  |  |  |
|  | *Country report* | 2,571 | 80.5 | 194 | 15.9 | 2,765 | 62.6 |
|  | *Published* | 604 | 18.9 | 1,017 | 83.1 | 1,621 | 36.7 |
|  | *Thesis/Book* | 20 | 0.6 | 13 | 1.1 | 33 | 0.8 |
| Type of survey |  |  |  |  |  |  |  |
|  | *LQAS* | 911 | 28.5 | 23 | 1.9 | 934 | 21.1 |
|  | *Mapping/prevalence* | 2,259 | 70.7 | 1,042 | 85.1 | 3,301 | 74.7 |
|  | *SS/Sport check* | 25 | 0.8 | 159 | 13.0 | 184 | 4.2 |
| Blood volume |  |  |  |  |  |  |  |
|  | *20-40µl* | - | 0.0 | 861 | 70.3 | 861 | 19.5 |
|  | *50-60 µl* | - | 0.0 | 196 | 16.0 | 196 | 4.4 |
|  | *100-120 µl* | 3,195 | 100.0 | 111 | 9.1 | 3,306 | 74.8 |
|  | *>200 µl* | - | 0.0 | 46 | 3.8 | 46 | 1.0 |
|  | *Unknown* | - | 0.0 | 10 | 0.8 | 10 | 0.2 |
| Age ranges examined | |  |  |  |  |  |  |
|  | *Unknown* | 461 | 14.4 | 68 | 5.6 | 529 | 12.0 |
|  | *≤ 15 years* | 76 | 2.4 | 5 | 0.4 | 81 | 1.8 |
|  | *16+ years* | 2,576 | 80.6 | 238 | 19.4 | 2,814 | 63.7 |
|  | *All ages* | 82 | 2.6 | 913 | 74.6 | 995 | 22.5 |
| Sex of people examined | |  |  |  |  |  |  |
|  | *Unknown* | 49 | 1.5 | 12 | 1.0 | 61 | 1.4 |
|  | *Males* | - | 0.0 | 44 | 3.6 | 44 | 1.0 |
|  | *Females* | - | 0.0 | - | 0.0 | - | 0.0 |
|  | *Both* | 3,146 | 98.5 | 1,168 | 95.4 | 4,314 | 97.6 |
|  |  |  |  |  |  |  |  |
|  |  | **median** | **IQR** | **median** | **IQR** | **median** | **IQR** |
| People examined | | 100 | 64-100 | 145 | 86-389 | 100 | 75-101 |

Source of data available by country at the website of the Global Atlas of Helminth Infections project ([www.thiswormyworld.org](http://www.thiswormyworld.org))

**Standardization of mf prevalence based on blood volume**

Previous studies have suggested that the sensitivity of methods for detecting LF microfilariae (mf) vary according to the quantity of blood sampled, especially in settings where mf load is low [1]. Therefore, some authors proposed standardizing mf prevalence values obtained from different protocols to reflect a 1ml blood sample, using transformation factors of 1.95 for 20µl and 1.15 for 100µl samples [2]. Such adjustment factors were based on four studies for 20µl and one study for 100µl samples [1, 3-6]. To examine this issue further, Table 1S presents the number of surveys conducted in the African region included in the global LF database which are based on different blood volumes and diagnostic method.

**Table 1S. Blood samples and diagnostic methods of surveys included in the global LF database (African region)**

| **Blood sample volume** | **Pre-intervention**  **N (%)** | **Post-intervention**  **N (%)** |
| --- | --- | --- |
| Blood smear |  |  |
| 20-40µl | 1,143 (22.4) | 283 (36.8) |
| 50-60 µl | 250 (4.9) | 288 (37.4) |
| 100-120 µl | 115 (2.2) | 36 (4.7) |
| Concentration | 58 (1.1) | 20 (2.6) |
| ICT | 3,522 (69.1) | 142 (18.5) |
| Total | 5,098 | 769 |

To decide if and how to adjust for blood volumes used for the estimation of mf prevalence, we identified 34 studies conducted in the African region before the implementation of large-scale MDA-based interventions which compared the different blood volumes and diagnostic methods (Table 2S)

**Table 2S. Paired estimates of LF prevalence based on blood smear and filtration methods**

| **Blood volume, µl** | **N** | **Estimated prevalence** ^a^ | | **p-value** ^b^ |
| --- | --- | --- | --- | --- |
|  |  | **Blood smear** | **Filtration** |  |
| 20 | 21 | 4.3 (0.5-12.3) | 11.8 (5.3-30.2) | <0.001 |
| 40 | 3 | 5.0 (4.7-11.6) | 11.6 (8.8-11.6) | 0.207 |
| 50 | 5 | 3.6 (1.0-5.6) | 7.5 (7.4-17.2) | 0.037 |
| 60 | 5 | 11.6 (5.4-24.7) | 18.1 (11.6-54.7) | 0.035 |

^a^ Median (25^th^-75^th^ percentile)

^b^ p-value based on paired Student t-test after empirical log transformation of mf prevalence

There is statistical evidence for a difference in prevalence estimates based on blood smear and filtration techniques (Table 2S). However, we were unable to derive adjustment terms of good quality that are representative for a range of transmission settings considering the limited availability of comparable data and the small proportion of mf prevalence data derived from filtration compared to all other methods. Moreover, sufficient data comparing directly blood smear mf prevalence based on different blood amounts are not available to adjust accurately between 20µl and other amounts used in blood smear (e.g. 60µl).

**References**

1. Sabry M. A quantitative approach to the relationship between Wuchereria bancrofti microfilaria counts by venous blood filtration and finger-prick blood films. Trans R Soc Trop Med Hyg. 1991;85(4):506-10.

2. Michael E, Malecela MN, Zervos M, Kazura JW. Global eradication of lymphatic filariasis: the value of chronic disease control in parasite elimination programmes. PloS One. 2008;3(8):e2936. doi:10.1371/journal.pone.0002936.

3. Desowitz RS, Southgate BA. Studies of filariasis in the Pacific. 2. The persistence of microfilaraemia in diethylcarbamazine treated populations of Fiji and Western Samoa: diagnostic application of the membrane-filtration technique. Southeast Asian J Trop Med Public Health. 1973;4(2):179-83.

4. McMahon JE, Marshall TF, Vaughan JP, Abaru DE. Bancroftian filariasis: a comparison of microfilariae counting techniques using counting chamber, standard slide and membrane (nuclepore) filtration. Ann Trop Med Parasitol. 1979;73(5):457-64.

5. Moulia-Pelat JP, Glaziou P, Nguyen-Ngoc L, Cardines D, Spiegel A, Cartel JL. A comparative study of detection methods for evaluation of microfilaremia in lymphatic filariasis control programmes. Trop Med Parasitol. 1992;43(3):146-8.

6. Dreyer G, Pimentael A, Medeiros Z, Beliz F, Moura I, Coutinho A et al. Studies on the periodicity and intravascular distribution of Wuchereria bancrofti microfilariae in paired samples of capillary and venous blood from Recife, Brazil. Trop Med Int Health. 1996;1(2):264-72.
