# Supplementary material for: Modelling the distribution and transmission intensity of lymphatic filariasis in sub-Saharan Africa prior to scaling up interventions: integrated use of geostatistical and mathematical modelling
Source: Parasit Vectors. 2015 Oct 24;8:560. doi: 10.1186/s13071-015-1166-x (PMC4620019; doi:10.1186/s13071-015-1166-x)
Supplement: Additional file 2: — Environmental and demographic covariates used in the geostatistical modelling. Table S3. Description of the covariates explored to model the microfilaraemia and antigenaemia prevalence. Table S4. Correlation matrix of environmental and demographic covariates (continuous data) included in the modelling. Spearman’s correlation test was used to explore the colinearity between pairs of covariates. Table S5. Relationship between infection risk and each potential explanatory variable. (DOCX 34 kb) [file 13071_2015_1166_MOESM2_ESM.docx]

**Additional file 2: Environmental and demographic covariates used in the geostatistical modelling**

**Table 3S.** Description of the covariates explored to model the microfilaraemia and antigenaemia prevalence.

| **Covariate** | **Description** | **Source of data** |
| --- | --- | --- |
| Annual Potential Evapo-transpiration | Potential Evapo-Transpiration (PET) is a measure of the ability of the atmosphere to remove water through Evapo-Transpiration (ET) processes. Global PET has been modeled using the data available from the WorldClim Global Climate Data as input parameters. | <http://www.cgiar-csi.org/data/global-aridity-and-pet-database> |
| Aridity Index | Indicator of the degree of dryness of the climate at a given location, and result from dividing the mean annual precipitation by the mean annual potential evapo-transpiration |  |
| Annual Accumulative precipitation | Mean of annual precipitation across the period 1950-2000 | <http://www.worldclim.org/bioclim> |
| Precipitation in the driest quarter | Total precipitation in the driest quarter (period 1950-2000) |  |
| Precipitation in the wettest quarter | Total precipitation in the wettest quarter (period 1950-2000) |  |
| Annual mean temperature | Mean of average monthly mean temperature across all 12 months (period 1950-2000) |  |
| Annual average of maximum temperature | Mean of average monthly maximum temperature across all 12 months (period 1950-2000) |  |
| Annual average of minimum temperature | Mean of average monthly minimum temperature across all 12 months (period 1950-2000) |  |
| Mean temperature in the coldest quarter | Mean of average monthly mean temperature in the coldest quarter (period 1950-2000) |  |
| Mean temperature in the warmest quarter | Mean of average monthly mean temperature in the warmest quarter (period 1950-2000) |  |
| Average of long-term measurement of Land Surface Temperature (MODIS-AVHRR) | Average of montly land surface temperature (LST) for the period July 2002 and June 2012. Daily measures of land surface temperature are obtained by the Moderate Resolution Imaging Spectoradiometer (MODIS). | ftp://africagrids.net/1000m/MYD11A2/ |
| Population density (according to the year of survey) | Population density 1960 | http://na.unep.net/siouxfalls/datasets/datalist.php |
|  | Population density 1970 |  |
|  | Population density 1980 |  |
|  | Population density 1990 |  |
|  | Population density 2000 |  |
|  | Population density 2010 | <http://www.worldpop.org.uk/> |
| Population Growth Rate (1960-2010) | The rate at which the number of individuals in a population increases in a given time period, expressed as a fraction of the initial population. | Obtained from population density estimates at 1960 and 2010 |
| Average of long-term measurement of Enhanced Vegetation Index (EVI) | Average of montly enhanced vegetation index (EVI) for the period January 2000 and June 2012. Monthly measures of EVI are obtained by the Moderate Resolution Imaging Spectoradiometer (MODIS). | ftp://africagrids.net/250m/MOD13Q1/ |
| Average of long-term measurement of Normalized Difference Vegetation Index (NDVI) | Average of montly normalized difference vegetation index (NDVI) for the period January 2000 and June 2012. Monthly measures of NDVI are obtained by the Moderate Resolution Imaging Spectoradiometer (MODIS). | ftp://africagrids.net/250m/MOD13Q1/ |
| Global Land Cover 2000 | Developed by the Joint Research Centre, European Comission. The GLC2000 project uses the FAO Land Cover Classification System (LCCS). | <http://bioval.jrc.ec.europa.eu/products/glc2000/data_access.php> |
| Reclassified raster obtained from Global Land Cover 2000 | Classes grouped into major groups; forest, woodlands, shrub lands and grasslands, agriculture, bare soil, water bodies and cities |  |
| Global Land Cover 2004-2006 | Developed by the Global Land Cover Network (FAO) | <http://www.glcn.org/index_en.jsp> |
| Reclassified raster obtained from Global Land Cover 2004-2006 | Classes grouped into major groups; forest, woodlands, shrub lands and grasslands, agriculture, bare soil, snow/ice, water bodies and cities |  |
| Global Land Cover 2009 | Developed by the Global Land Cover Network (FAO) |  |
| Reclassified raster obtained from Global Land Cover 2009 | Classes grouped into major groups; forest, woodlands, shrub lands and grasslands, agriculture, bare soil, snow/ice, water bodies and cities |  |
| Euclidean distance to water bodies | Straight line distance to the nearest water body. A gridded map of water bodies was obtained from World Wild Life organization (WWF). The Hydrology level 3 product comprises lakes, reservoirs, rivers and different wetland types in the form of a global raster map at 30-second resolution. | <http://worldwildlife.org/pages/conservation-science-data-and-tools> |
| Euclidean distance to rivers | Straight line distance to the nearest stream. A vector layer of rivers was obtained from the Digital Chart of the World. | <http://www.diva-gis.org/gdata> |
| SRTM Digital Elevation Data - 1 km | Resampled DEM at 1 km resolution | <http://www.cgiar-csi.org/data/srtm-90m-digital-elevation-database-v4-1> |
| Slope based on the DEM at 1 km | Slope provided in degrees |  |
| Urban, rural and peri-urban areas from pop density 1960 | These categorical rasters have been obtained from population density surfaces based on the following criteria: urban extents (UE) have a population densities ≥1,000 inhab/km^2^, peri-urban >250 inhab /km^2^ within a 15 km distance from UE edge, and rural <250 inhab/km^2^ and/or >15 km from the UE edge |  |
| Urban, rural and peri-urban areas from pop density 1970 |  |  |
| Urban, rural and peri-urban areas from pop density 1980 |  |  |
| Urban, rural and peri-urban areas from pop density 1990 |  |  |
| Urban, rural and peri-urban areas from pop density 2000 |  |  |
| Urban, rural and peri-urban areas from pop density 2010 |  |  |
| Ecological Zones according to FAO | A globally consistent classification has been adopted, based on the Koppen-Trewartha climate system in combination with natural vegetation characteristics. A total of 19 global ecological zones have been defined and mapped | <http://www.fao.org/geonetwork/srv/en/main.home#biological> |
| Predicted distribution of Anopheles fuestus/gambiae complex in Africa | Based on Boosted regression trees environmental model. Obtained from Malaria Atlas project | <http://www.map.ox.ac.uk/> |
| Predicted distribution of Culex mosquitoes in Africa | Maxent based approach (ecological modelling based on maximum entropy approach) | <http://www.vectormap.org/> |
| Predicted distribution of Mansonia africana |  |  |
| Zonation based on the distribution of major potential LF vectors | This raster surface has been obtained from overlapping predictive models of vector distribution; distribution of Anopheles gambiae and funestus complex (based on BRT modelling) and Culex pipiens, Cx quinquefasciatus and Mansonia african maps, based on Maxent approach |  |

**Table 4S.** Correlation matrix of environmental and demographic covariates (continuous data) included in the modelling. Spearman’s correlation test was used to explore the colinearity between pairs of covariates.

|  | AnPET | AI | AAcP | PDQ | PWQ | AMT | AMxT | AMiT | LST | TCQ | TWQ | PD | PGR | EVI | NDVI | DistWB | DistSt | Slope | DEM |
| --- | --- | --- | --- | --- | --- | --- | --- | --- | --- | --- | --- | --- | --- | --- | --- | --- | --- | --- | --- |
| AnPET | 1.00 |  |  |  |  |  |  |  |  |  |  |  |  |  |  |  |  |  |  |
| AI | -0.44 | 1.00 |  |  |  |  |  |  |  |  |  |  |  |  |  |  |  |  |  |
| AAcP | -0.29 | 0.98 | 1.00 |  |  |  |  |  |  |  |  |  |  |  |  |  |  |  |  |
| PDQ | -0.35 | 0.64 | 0.61 | 1.00 |  |  |  |  |  |  |  |  |  |  |  |  |  |  |  |
| PWQ | -0.17 | 0.87 | 0.89 | 0.30 | 1.00 |  |  |  |  |  |  |  |  |  |  |  |  |  |  |
| AMT | 0.61 | -0.04 | 0.06 | -0.07 | 0.07 | 1.00 |  |  |  |  |  |  |  |  |  |  |  |  |  |
| AMxT | 0.81 | -0.22 | -0.09 | -0.20 | -0.03 | 0.95 | 1.00 |  |  |  |  |  |  |  |  |  |  |  |  |
| AMinT | 0.40 | 0.12 | 0.19 | 0.04 | 0.15 | 0.96 | 0.85 | 1.00 |  |  |  |  |  |  |  |  |  |  |  |
| LST | 0.67 | -0.65 | -0.60 | -0.54 | -0.37 | 0.31 | 0.48 | 0.15 | 1.00 |  |  |  |  |  |  |  |  |  |  |
| TCQ | 0.55 | 0.17 | 0.27 | 0.07 | 0.25 | 0.91 | 0.84 | 0.91 | 0.22 | 1.00 |  |  |  |  |  |  |  |  |  |
| TWQ | 0.62 | -0.23 | -0.15 | -0.21 | -0.09 | 0.93 | 0.93 | 0.88 | 0.40 | 0.75 | 1.00 |  |  |  |  |  |  |  |  |
| PD | -0.08 | -0.09 | -0.11 | -0.04 | -0.14 | -0.12 | -0.10 | -0.13 | 0.00 | -0.17 | -0.07 | 1.00 |  |  |  |  |  |  |  |
| PGR | 0.01 | 0.08 | 0.09 | 0.05 | 0.08 | -0.01 | -0.02 | 0.00 | -0.03 | 0.06 | -0.08 | -0.03 | 1.00 |  |  |  |  |  |  |
| EVI | -0.37 | 0.55 | 0.55 | 0.53 | 0.32 | 0.03 | -0.12 | 0.16 | -0.76 | 0.11 | -0.08 | -0.07 | 0.05 | 1.00 |  |  |  |  |  |
| NDVI | -0.38 | 0.51 | 0.50 | 0.52 | 0.28 | -0.07 | -0.21 | 0.04 | -0.72 | 0.01 | -0.18 | -0.09 | 0.10 | 0.78 | 1.00 |  |  |  |  |
| DistWB | 0.11 | -0.09 | -0.07 | -0.08 | -0.04 | -0.12 | -0.05 | -0.16 | 0.11 | -0.07 | -0.13 | -0.02 | 0.01 | -0.10 | -0.10 | 1.00 |  |  |  |
| DistSt | 0.07 | -0.02 | -0.02 | -0.01 | 0.00 | 0.09 | 0.09 | 0.08 | 0.09 | 0.11 | 0.06 | -0.03 | 0.03 | -0.07 | -0.13 | 0.14 | 1.00 |  |  |
| Slope | -0.28 | 0.11 | 0.07 | 0.06 | 0.08 | -0.53 | -0.51 | -0.52 | -0.13 | -0.40 | -0.56 | 0.02 | 0.05 | -0.05 | 0.00 | 0.17 | -0.02 | 1.00 |  |
| DEM | -0.31 | 0.04 | -0.01 | 0.02 | 0.02 | -0.84 | -0.76 | -0.87 | -0.09 | -0.64 | -0.90 | 0.04 | 0.08 | -0.19 | -0.07 | 0.21 | -0.02 | 0.61 | 1.00 |

AnPET: annual potential evapo-transpiration; AI: aridity index; AAcP: annual precipitation; PDQ: precipitation driest quarter; PWQ: precipitation wettest quarter; AMT: annual mean temperature; AMxT: annual maximum temperature; AMiT: annual minimum temperature; LST: land surface temperature; TCQ: temperature in the coldest quarter; TWQ: temperature in the warmest quarter; PD: population density; PGR: population growth rate; EVI: enhanced vegetation index; NDVI: normalized difference vegetation index; DistWB: distance to water bodies; DistSt: distance to streams; DEM: altitude

**Table 5S.** Relationship between infection risk and each potential explanatory variable.

Variables were grouped according to its nature and collinearity. Selection of variables was made by fitting univariate models relating the logit of infection to each of the covariate and eventually comparing the models in terms of the Akaike Information Criterion (AIC), choosing those variables which yielded the lowest AIC value. Highlighted in grey the covariates selected to fit the geostatistical models. Some variables were finally dropped when running the final geostatistical model based on antigenaemia prevalence (*).

|  |  | **AIC value** | |
| --- | --- | --- | --- |
| **Group** | **Group of covariates** | **pmf** | **pICT** |
| 1 | *Age range* | 43,048.63 | 41,368.04 |
| 2 | *Period of time^&^* | 44,063.15 | - |
| 3 | *Land cover 2000* | 42,407.48 | 67,590.88 |
|  | *Land cover 2004-2006* | 42,731.11 | 69,955.69 |
|  | *Land cover 2009* | 42,651.49 | 70,123.64 |
| 4 | *Urbanization* | 43,646.30 | 68,051.23 |
| 5 | *Aridity Index* | 41,395.35 | 69,680.67 |
|  | *Annual Precipitation* | 41,282.92 | 69,226.96 |
|  | *Precipitation Wettest Quarter* | 41,206.24 | 67,987.03 |
| 6 | *Precipitation Driest Quarter* | 41,444.16 | 70,241.65 |
| 7 | *Mean temperature* | 39,044.12 | 55,804.99 |
|  | *Maximum temperature* | 39,108.56 | 59,489.12 |
|  | *Minimum temperature* | 38,665.86 | 53,617.73 |
|  | *Temperature coldest quarter* | 38,967.98 | 59,385.06 |
|  | *Temperature warmest quarter* | 39,068.98 | 53,598.03 |
|  | *Potential Evapo-transpiration* | 38,897.56 | 68,295.00 |
|  | *Elevation* | 38,344.77 | 51,088.54 |
| 8 | *Land Surface temperature* | 38,759.78 | 68,191.49* |
| 9 | *Population growth rate* | 39,250.97 | 70,191.89 |
| 10 | *EVI* | 39,030.52 | 69,419.11 |
|  | *NDVI* | 39,070.52 | 69,763.38 |
| 11 | *Distance to water bodies* | 42,186.79 | 69,662.08* |
|  | *Distance to streams* | 42,108.16 | 70,227.28 |
| 12 | *Slope* | 41,665.84 | 63,484.01* |
| 13 | *Population density* | 43,078.70 | 69,149.24 |
| 14 | *Anopheles spp distribution* | 44,128.54 | 70,683.28 |
| 15 | *Culex spp distribution* | 43,870.85 | 70,713.40 |
| 16 | *Mansonia africana distribution* | 45,061.74 | 57,936.25 |

^&^ Period of time at which the mf-surveys were undertaken
